# Estimated Pulse Wave Velocity in the Prediction of Clinical Outcomes in Patients Undergoing Drug-Eluting Stent Implantation

**DOI:** 10.3390/jcm12185855

**Published:** 2023-09-08

**Authors:** Hack-Lyoung Kim, Hyun Sung Joh, Woo-Hyun Lim, Jae-Bin Seo, Sang-Hyun Kim, Joo-Hee Zo, Myung-A Kim

**Affiliations:** Division of Cardiology, Department of Internal Medicine, Seoul Metropolitan Government-Seoul National University Boramae Medical Center, Seoul National University College of Medicine, Seoul 07061, Republic of Korea; wingx4@naver.com (H.S.J.); woosion@gmail.com (W.-H.L.);

**Keywords:** arterial stiffness, cardiovascular disease, percutaneous coronary intervention, prognosis, pulse wave analysis

## Abstract

Background The prognostic value of estimated pulse wave velocity (ePWV) has been infrequently explored in high-risk patient groups. Our study aimed to evaluate the prognostic significance of ePWV among patients undergoing a percutaneous coronary intervention (PCI) with a drug-eluting stent (DES). Methods A total of 4119 consecutive subjects who underwent a PCI with a DES (mean age, 67.1 ± 11.6 years and 33.1% were female) were retrospectively analyzed. ePWV was calculated based on the patient’s age and mean blood pressure. Major adverse cardiovascular events (MACE), including cardiac death, non-fatal myocardial infarction, coronary revascularization, and ischemic stroke, were evaluated. Results During a median follow-up duration of 3.51 years (interquartile range, 1.35–6.37 years), there were 746 MACEs (18.1%). A multivariable analysis showed that a higher ePWV was associated with a higher MACE incidence (middle tertile vs. the lowest tertile: hazard ratio [HR], 2.49; 95% confidence interval [CI], 1.81–3.42; *p* < 0.001; the highest tertile vs. the lowest tertile: HR, 6.18; 95% CI, 4.33–8.80; *p* < 0.001) The inclusion of ePWV data significantly increased the global chi-square values when added to the clinical information (from 96 to 128; *p* < 0.001). Conclusion ePWV demonstrated a significant association with MACEs in patients who underwent DES implantation. Given its relative simplicity to calculate, ePWV could potentially serve as a valuable instrument for stratifying cardiovascular risks within this high-risk patient population.

## 1. Introduction

Coronary Artery Disease (CAD) remains a leading cause of mortality worldwide, despite significant advances in medical science [1]. Characterized by a blockage of the coronary arteries, CAD can lead to debilitating health complications such as myocardial infarction and sudden cardiac death [2]. Modern treatments have enhanced its short-term survival rates, but there is room for improvement in its long-term prognosis, particularly given the chronic and progressive characteristics of the disease [1]. This situation underscores the essential need for an effective prognostic tool for CAD. Such a tool would provide a more precise risk assessment, enabling timely interventions, personalized treatment strategies, and, ultimately, better patient outcomes.

Arterial stiffening refers to a reduction in the elasticity of arteries, typically caused by aging and unmanaged risk factors such as hypertension, hyperglycemia, dyslipidemia, smoking, inflammation, and oxidative stress [3,4]. Increased arterial stiffness augments cardiovascular load and adversely affects cardiac function, making it a key factor in cardiovascular disease progression, including CAD [5,6,7]. A growing body of evidence suggests that arterial stiffness, measured using methods such as pulse wave velocity (PWV), can serve as a significant predictive marker for CAD prognosis [6,8,9,10]. Understanding and monitoring arterial stiffness could thus become an integral part of CAD prognosis prediction.

Pulse Wave Velocity (PWV) is a clinical indicator of arterial stiffness, reflecting the rate at which pressure waves traverse the arteries [4]. Traditionally, PWV is determined by dividing the distance between two specific points by the time difference of pulse wave arrival using specialized equipment. However, it can also be estimated based on age and blood pressure (BP), the primary factors known to influence arterial elasticity [11,12,13,14]. In this study, we utilized this “estimated PWV (ePWV)” as a prognostic tool for patients who had undergone a PCI. The aim was to investigate whether ePWV, derived from simple, readily available clinical parameters, could provide a reliable prediction of long-term outcomes post-PCI. This could potentially enhance personalized care and improve health outcomes for CAD patients.

## 2. Materials and Methods

### 2.1. Study Design and Population

This study is a retrospective analysis conducted at the cardiovascular center of Boramae Medical Center, a general hospital situated in Seoul, Republic of Korea. Between August 2008 and June 2020, we examined a total of 4270 consecutive patients who visited the center and underwent successful drug-eluting stent (DES) implantation for conditions such as angina pectoris or myocardial infarction. Following the exclusion of in-hospital mortality cases (*n* = 151), a total of 4119 patients were included in the final analysis. The study protocol was approved by the Institutional Review Board (IRB) of Boramae Medical Center. Due to the retrospective nature of the study, the IRB waived the requirement for obtaining informed consent.

### 2.2. Clinical Data Collection

Body mass index (BMI) was calculated by dividing an individual’s weight in kilograms by the square of their height in meters. Hypertension was identified if any of the following criteria were met: a prior diagnosis of hypertension by a physician, ongoing anti-hypertensive medication use, or consistent systolic/diastolic BP readings above 140/90 mmHg. Diabetes mellitus was similarly identified based on any of the following: a prior diagnosis by a physician, current use of anti-diabetic medication, repeated fasting blood glucose levels of ≥126 mg/dL, or at least one instance of a glycated hemoglobin level of ≥6.5%. Current smoking status was determined by regular and active smoking within the preceding year. Acute myocardial infarction was diagnosed based on chest pain, electrocardiographic alterations, troponin elevation, and angiographic findings. Previous CAD was determined by a history of myocardial infarction or coronary revascularization. After overnight fasting, venous blood samples were drawn from the antecubital vein to measure parameters such as hemoglobin, total cholesterol, low-density lipoprotein cholesterol, high-density lipoprotein cholesterol, triglycerides, creatinine, glycated hemoglobin, uric acid, and C-reactive protein. The glomerular filtration rate (GFR) was estimated using the equation from the Modification of Diet in Renal Disease study. Left ventricular ejection fraction was acquired through transthoracic echocardiography using the biplane method. Additionally, data regarding the use of cardiovascular medications at discharge, including beta-blockers, renin-angiotensin system blockers, and statins, were collected.

### 2.3. Invasive Coronary Angiography and PCI Procedure

Invasive coronary angiography (ICA) was conducted in line with the standard guideline recommendations [15]. A luminal stenosis exceeding 50% in the epicardial coronary artery was considered to be an obstructive lesion. The terms one-vessel disease, two-vessel disease, and three-vessel disease were used to describe the condition based on the number of epicardial coronary arteries exhibiting stenotic lesions beyond 50%. A 50% or more stenosis in the left main coronary artery was classified as two-vessel disease. The decision to undergo PCI was based on the patient’s symptoms, stress test outcomes, and ICA results, at the discretion of the supervising cardiologist. The PCI procedure was executed following the current international guidelines [15].

### 2.4. ePWV Calculation

BP was measured using an automatic sphygmomanometer on the right upper arm while the patient was in a stable sitting position during their hospital stay. The average systolic BP (SBP) and diastolic BP (DBP) were calculated by taking the mean of three measurements obtained just prior to the patient’s stable discharge. The mean BP (MBP) was determined using the formula: MBP = DBP + [0.4 × (SBP-DBP)]. The ePWV was computed using the patient’s age and MBP according to the following equation: ePWV (m/s) = 9.587 − (0.402 × age) + (4.560 × 0.001 × age^2^) − (2.621 × 0.00001 × age^2^ × MBP) + 3.176 × 0.001 × age × MBP) − (1.832 × 0.01 × MBP) [11].

### 2.5. Clinical Outcome

Details regarding major adverse cardiovascular events (MACE) were evaluated during the clinical follow-up after the PCI. The MACE information was primarily obtained from medical record reviews. For patients who were lost to clinical follow-up for more than 6 months, we used telephone interviews and national death records. MACEs encompassed cardiac death, non-fatal myocardial infarction, coronary revascularization, and ischemic stroke. Cardiac death was characterized as death resulting from acute coronary syndrome, fatal ventricular arrhythmia, or heart failure. Unexplained sudden death was likewise classified under cardiac death. A myocardial infarction was recognized based on symptoms such as chest pain, elevated troponin levels, electrocardiographic changes, and the presence of obstructive coronary lesions, as detected using coronary angiography. Coronary revascularization included procedures such as PCI and coronary bypass surgery. Ischemic stroke was confirmed based on the sudden onset of neurological symptoms, verified by detecting areas of infarction in brain imaging studies. If multiple events were experienced simultaneously, the first event was categorized as the MACE. Events occurring within 30 days from the date of the PCI procedure were attributed to the underlying condition necessitating the intervention, and were thus not classified as MACE.

### 2.6. Statistical Analysis

Continuous variables are shown as mean ± standard deviation, while categorical variables are expressed as numbers (percentages). The Student *t*-test and chi-square test were used to compare the continuous and categorical variables between patients with and without MACE, respectively. For grouping, the median value and tertiles of ePWV were utilized. The MACE incidence in relation to the ePWV tertiles was assessed using the chi-square test for linear association. The receiver operating characteristic (ROC) curve analysis determined the optimal ePWV cut-off for MACE prediction. Multivariable Cox regression analyses established the independent relationship between ePWV and MACEs, adjusting for various clinical covariates such as age, sex, body mass index, hypertension, diabetes mellitus, prior history of CAD, acute myocardial infarction diagnosis, CAD severity, smoking, glomerular filtration rate, left ventricular ejection fraction, and the usage of beta-blockers and renin-angiotensin system blockers. The Kaplan–Meier survival curve analysis was applied to demonstrate the prognostic significance of ePWV, and the log-rank test was used for statistical significance. The incremental predictive value of ePWV over clinical factors (age, sex, body mass index, hypertension, diabetes mellitus, previous CAD, diagnosis of acute myocardial infarction, cigarette smoking, glomerular filtration rate, and the use of beta-blockers and renin-angiotensin system blockers) for MACE prediction was evaluated by assessing changes in global chi-squares [16,17]. A significant chi-square value suggested that the model with predictors provided a better fit to the data than the model without predictors. All the analyses were two-tailed, with a *p*-value less than 0.05, denoting statistical significance. All the statistical computations were performed using SPSS version 23.0 (IBM Co., Armonk, NY, USA).

## 3. Results

### Baseline Clinical Characteristics in Patients with and without MACE

Throughout a median follow-up period of 3.51 years (interquartile range, 1.35~6.37 years), a total of 746 cases of MACEs (18.1%) were reported. These MACEs included 100 cases of cardiac death (2.4%), 102 non-fatal myocardial infarctions (2.4%), 530 instances of coronary revascularization (12.8%), and 114 occurrences of ischemic stroke (2.7%).

Table 1 presents a comparative analysis of the baseline clinical characteristics between the patients who experienced a MACE and those who did not. The patients who experienced a MACE were generally older (69.1 ± 11.4 vs. 66.5 ± 11.5 years; *p* < 0.001). Both groups had similar gender proportions (female: 32.9% vs. 33.2%) and comparable body mass indices (*p* > 0.05 for each). Compared to the patients who did not experience a MACE, those who did had a more significant number of cardiovascular risk factors, including hypertension, diabetes mellitus, and prior CAD. The laboratory findings revealed lower levels of hemoglobin and high-density lipoprotein cholesterol, and higher levels of low-density lipoprotein cholesterol, glomerular filtration rate, glycated hemoglobin, uric acid, and C-reactive protein in the MACE group. The MACE group also had a lower left ventricular ejection fraction (57.9% ± 14.8% vs. 60.3% *±* 12.4%; *p* < 0.001) and more severe CAD, as evidenced by a higher prevalence of three-vessel disease (55.1% vs. 37.3%, *p* < 0.001). No significant differences were observed in the discharge medications between the two groups.

**Figure 1:**
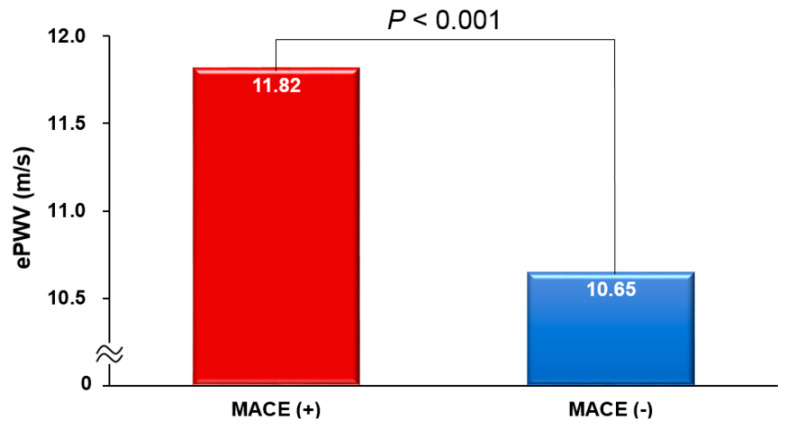
ePWV difference between patients with and without MACE. ePWV, estimated pulse wave velocity; and MACE, major adverse cardiovascular event.

**Figure 2:**
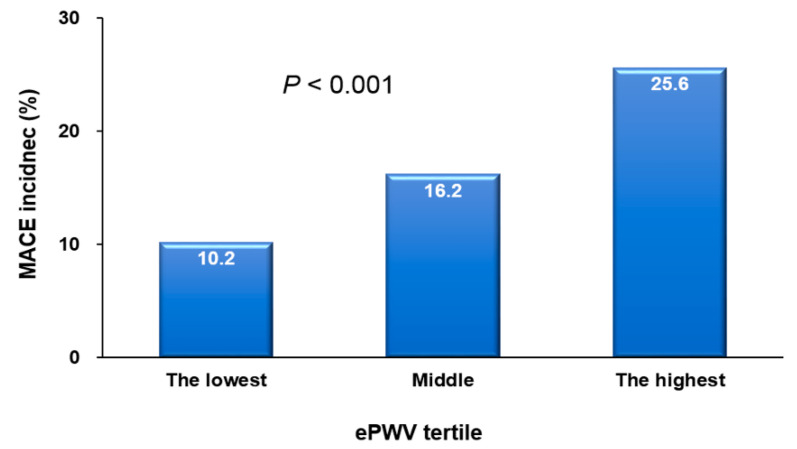
MACE incidence according to ePWV tertile. MACE, major adverse cardiovascular event; and ePWV, estimated pulse wave velocity.

The ePWV was notably higher in the patients who experienced a MACE compared to those who did not (11.82 ± 2.13 vs. 10.65 ± 2.13 m/s; *p* < 0.001) (Figure 1). There was a proportional increase in the incidence of MACEs from the lowest to the highest tertile of the ePWV (*p* < 0.001) (Figure 2).

**Figure 3:**
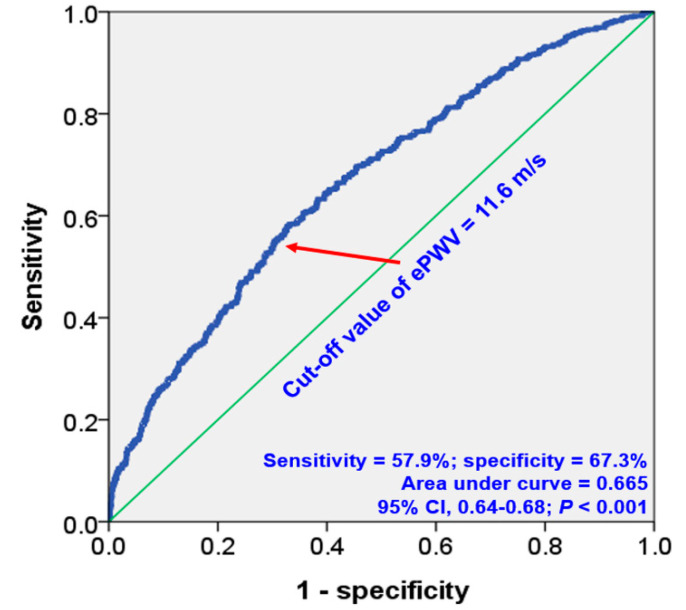
ROC curve analysis showing cut-off value of ePWV in the prediction of MACEs. ROC, receiver operating characteristic; MACE, major adverse cardiovascular event; and CI, confidence interval.

The ROC curve analysis identified an ePWV cut-off value of 11.6 m/s for predicting MACEs, yielding a sensitivity of 57.6% and a specificity of 67.3% (area under curve, 0.665; 95% confidence interval, 0.64–0.68; *p* < 0.001) (Figure 3).

**Figure 4:**
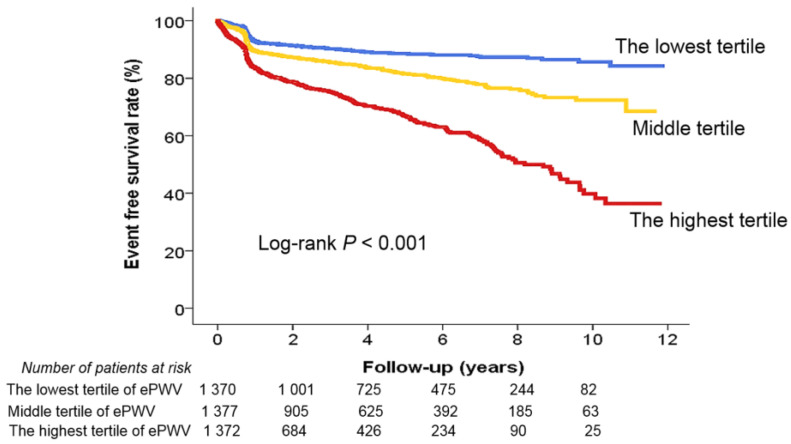
Event-free survival rate according to ePWV tertile. ePWV, estimated pulse wave velocity.

The multivariable analyses established that a higher ePWV served as a significant predictor for MACEs (Table 2). When the ePWV was divided into different thresholds—the median value (ePWV ≥ 10.9 m/s: hazard ratio [HR], 3.78; 95% confidence interval [CI]; 2.83–4.91; *p* < 0.001), the cut-off value from the ROC curve analysis (ePWV ≥ 11.6 m/s: HR, 3.56; 95% CI, 2.77–4.58; *p* < 0.001), and tertile criteria (middle tertile vs. the lowest tertile: HR, 2.49; 95% CI, 1.81–3.42; *p* < 0.001; the highest tertile vs. the lowest tertile: HR, 6.18; 95% CI, 4.33–8.80; *p* < 0.001)—a high ePWV under all conditions independently predicted MACEs. Old age, obesity, diabetes mellitus, more severe CAD, and renal and left ventricular dysfunction were also associated with MACEs (Supplementary Tables S1–S3). The Kaplan–Meier survival curve analysis further highlighted significant differences in the patients’ MACE risk across the ePWV tertiles (log-rank *p* < 0.001) (Figure 4).

**Figure 5:**
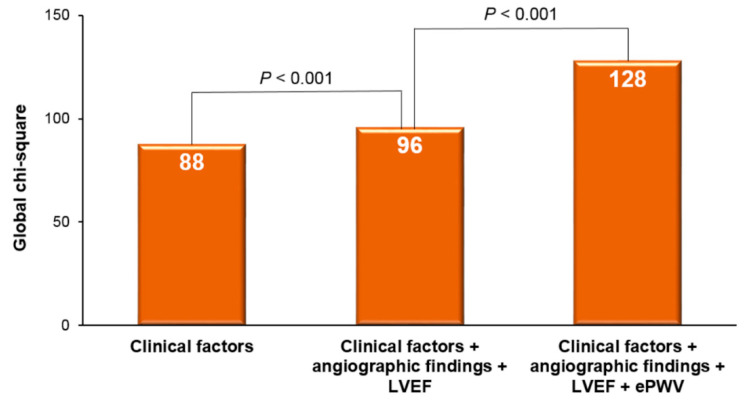
Incremental prognostic value of ePWV to clinical factors for the prediction of MACEs. MACE, major adverse cardiovascular event; LVEF, left ventricular ejection fraction; and ePWV, estimated pulse wave velocity.

The predictive power for MACEs increased significantly when the angiographic findings and left ventricular ejection fraction were added to the other clinical factors, as evidenced by a rise in the global chi-square from 88 to 96 (*p* < 0.001). Further inclusion of ePWV information to the clinical factors, angiographic findings, and left ventricular ejection fraction resulted in an even greater enhancement of MACE prediction, elevating the global chi-square from 96 to 128 (*p* < 0.001). These results are illustrated in Figure 5.

## 4. Discussion

The principal discovery of this study was the association between ePWV and MACE in patients who received DES implantation. As far as we know, this study is the first to confirm the prognostic significance of ePWV in patients undergoing a PCI.

Prior studies have published findings regarding the prognostic value of ePWV. In the general population, an elevated baseline ePWV has been linked to an increased likelihood of subsequent cardiovascular events or mortality [18,19,20]. Additionally, the prognostic value of ePWV in patients at a moderate or high risk has been revealed by some research. For example, one study involving 1040 individuals undergoing ICA found a significant association between ePWV and clinical outcomes, even after adjusting for potential confounders [8]. In the Systolic Blood Pressure Intervention Trial, which included 9361 participants with high risk profiles, an elevated ePWV was associated with cardiovascular risk, independently of Framingham Risk scores [12]. Further studies have underscored the prognostic value of ePWV in stroke patients [13] and those with diabetes mellitus [21]. Yet, the significance of ePWV for patients undergoing a PCI has not been firmly established. Given the high mortality rate associated with CAD, our study, which demonstrated the prognostic value of ePWV in CAD patients, is both novel and of great importance.

The mechanistic link between elevated arterial stiffness and a patient’s cardiovascular risk is well established [3,4]. A greater arterial stiffness, indicated by an elevated PWV, is associated with an increased likelihood of cardiovascular incidents and mortality across diverse patient groups. This is because arterial stiffness can cause an upsurge in systolic BP, augment cardiac afterload, leading to left ventricular hypertrophy, and inhibit coronary perfusion, all of which can precipitate cardiovascular events. Moreover, an escalated arterial stiffness shares cardiovascular risk factors such as hyperglycemia, dyslipidemia, inflammation, and oxidative stress [4]. As such, assessing arterial stiffness offers crucial insights into a patient’s vascular health and risks for future adverse occurrences [5].

The findings from this study have potential implications for patient care. Incorporating ePWV assessments into standard post-PCI evaluations could assist in early risk identification, expedite therapeutic intervention, and enhance the management of patients at a high risk for MACEs. The evidence pointing towards a substantial prognostic improvement by adding ePWV data to other clinical parameters, as revealed by this study, underscores the promising potential of this approach. Additionally, managing arterial stiffness could emerge as a vital therapeutic target. Lifestyle modifications, the management of BP, and specific medication may decrease arterial stiffness and lower cardiovascular risk. Moreover, the regular monitoring of arterial stiffness can provide valuable insights into these interventions’ effectiveness, thereby helping to optimize patient care. ePWV can be computed only if BP values are available, which could further enhance its economic utility. The practicality of routine ePWV measurements in various clinical settings will require further exploration in future research.

Despite ePWV being derived from Western data [11], as demonstrated in our study and several others, it appears to be highly relevant for Asian and Korean populations as well [17,18]. Through our research, we anticipate that ePWV could be applied to other patient groups or different ethnic populations.

### 4.1. Study Limitations

Our study has several limitations. Firstly, being a retrospective study, the incidence of MACEs may have been underestimated due to potential unrecognized occurrences. For similar reasons, we may not have accounted for all the major confounding variables. Secondly, we used BP measurements taken when the patient was stable at the time of discharge. Given that BP is a primary determinant of ePWV, accurate measurement methods are vital. Our study, however, being retrospective in nature, did not offer stringent guidelines for BP measurement. We attempted to mitigate this limitation by taking an average of three readings. Lastly, as our study population consisted of Koreans who underwent DES implantation, the results might not be transferable to other populations.

### 4.2. Study Conclusions

ePWV exhibited a significant association with MACEs in patients who underwent DES implantation. Given its ease of calculation, ePWV could potentially serve as a valuable tool for stratifying cardiovascular risks within this high-risk patient population.

## Figures and Tables

**Table 1:** Baseline clinical characteristics of study patients.

Characteristic	MACE (+) (*n* = 746)	MACE (−) (*n* = 3370)	*p*
Age, years	69.1 ± 11.4	66.5 ± 11.5	<0.001
Female sex	245 (32.9)	1118 (33.2)	0.888
Body mass index, kg/m^2^	24.3 ± 3.5	24.5 ± 3.4	0.452
Clinical diagnosis of myocardial infarction	340 (45.7)	1331 (39.5)	0.001
Risk factors			
Hypertension	490 (65.7)	2092 (62.1)	0.048
Diabetes mellitus	324 (43.5)	1112 (33.0)	<0.001
Current cigarette smoking	218 (29.3)	991 (29.4)	0.998
Previous coronary artery disease	341 (45.7)	1331 (39.5)	0.001
Laboratory findings			
Hemoglobin, g/dL	12.8 ± 2.1	13.2 ± 1.9	<0.001
Total cholesterol, mg/dL	165 ± 49	167 ± 44	0.282
Low-density lipoprotein cholesterol, mg/dL	99.1 ± 39.5	96.4 ± 36.7	0.217
High-density lipoprotein cholesterol, mg/dL	39.8 ± 11.2	41.9 ± 10.9	0.001
Triglyceride, mg/dL	134 ± 100	128 ± 76	0.251
Glomerular filtration rate, mL/min/1.73 m^2^	66.2 ± 29.7	76.8 ± 26.5	<0.001
Glycated hemoglobin, %	6.96 ± 1.62	6.57 ± 1.34	<0.001
Uric acid, mg/dL	5.85 ± 1.90	5.46 ± 1.89	<0.001
C-reactive protein, mg/dL	2.26 ± 5.38	1.29 ± 3.69	<0.001
Left ventricular ejection fraction, %	57.9 ± 14.8	60.3 ± 12.4	<0.001
Findings of invasive coronary angiography			<0.001
One-vessel disease	128 (17.2)	984 (29.2)	
Two-vessel disease	206 (27.7)	1128 (33.5)	
Three-vessel disease	411(55.1)	1287 (37.3)	
Medications			
Dual anti-platelets	746 (100)	3370 (100)	1.000
Calcium channel blockers	197 (26.3)	967 (28.7)	0.145
Renin-angiotensin system blockers	256 (34.4)	1118 (33.2)	0.480
Beta-blockers	240 (32.2)	1101 (32.7)	0.746
Statins	746 (100)	3370 (100)	1.000

Numbers are expressed as mean ± standard deviation or *n* (%). MACE, major adverse cardiovascular event.

**Table 2:** Multivariable analyses showing an independent association between ePWV and MACE.

Independent Variable	HR (95% CI)	*p*
ePWV ≥ 10.9 m/s (median value)	3.78 (2.83–4.91)	<0.001
ePWV ≥ 11.6 m/s (cut-off value)	3.56 (2.77–4.58)	<0.001
ePWV tertile		
The lowest tertile (5.50~9.89 m/s)	1	-
Middle tertile (9.91~11.98 m/s)	2.49 (1.81–3.42)	<0.001
The highest tertile (11.99~17.52 m/s)	6.18 (4.33–8.80)	<0.001

Separate multivariable models were used for each independent variable. The following clinical covariates were controlled as potential confounders during multivariable analyses: age, sex, body mass index, hypertension, diabetes mellitus, previous history of coronary artery disease, diagnosis of acute myocardial infarction, the severity of coronary artery disease, cigarette smoking, glomerular filtration rate, left ventricular ejection fraction, beta-blockers, and renin-angiotensin system blockers. ePWV, estimated pulse wave velocity; MACE, major adverse cardiovascular event; HR, hazard ratio; and CI, confidence interval.

## Data Availability

The data presented in this study are available on request from the corresponding author. The data are not publicly available due to privacy and ethical restrictions.

## Supplementary Materials

The following supporting information can be downloaded at: https://www.mdpi.com/article/10.3390/jcm12185855/s1, Table S1: Multivariable analysis showing the independent association between ePWV (≥10.9 m/s) and MACE; Table S2: Multivariable analysis showing the independent association between ePWV (≥11.6 m/s) and MACE; Table S3: Multivariable analysis showing the independent association between ePWV tertile and MACE.
