# Supplementary material for: Estimated Pulse Wave Velocity in the Prediction of Clinical Outcomes in Patients Undergoing Drug-Eluting Stent Implantation
Source: J Clin Med. 2023 Sep 8;12(18):5855. doi: 10.3390/jcm12185855 (PMC10531714; doi:10.3390/jcm12185855)
Supplement: Supplementary file 1 [file jcm-12-05855-s001.zip › jcm-2574251-SI.pdf]

Supplementary data

Supplementary Table S1. Multivariable analysis showing the independent association between ePWV (≥10.9 m/s) and MACE

**Independent variable**	**HR (95% CI)**	***P* value**
Age ≥ 65 years	1.78 (1.34-2.37)	<0.001
Female sex	0.86 (0.69-1.06)	0.159
Body mass index ≥ 25 kg/m^2	1.29 (1.06-1.56)	0.008
Hypertension	0.97 (0.78-1.21)	0.826
Diabetes mellitus	1.29 (0.96-1.61)	0.008
Previous coronary artery disease	1.24 (0.96-1.61)	0.094
Diagnosis of acute myocardial infarction	1.17 (0.94-1.46)	0.153
Number of obstructive epicardial arties	1.30 (1.15-1.47)	<0.001
Current cigarette smoking	1.24 (0.99-1.55)	0.055
Estimated GFR < 60 mL/min/1.73m^2	1.59 (1.29-1.97)	<0.001
Left ventricular ejection fraction < 55%	1.28 (1.03-1.60)	0.025
Beta-blockers	0.92 (0.76-1.12)	0.452
Renin-angiotensin system blockers	0.90 (0.74-1.09)	0.301
ePWV ≥ 10.9 m/s (median value)	3.78 (2.83-4.91)	<0.001

ePWV, estimated pulse wave velocity; MACE, major adverse cardiovascular event; GFR, glomerular filtration rate; HR, hazard ratio; CI, confidence interval.

Supplementary Table S2. Multivariable analysis showing the independent association between ePWV (≥11.6 m/s) and MACE

**Independent variable**	**HR (95% CI)**	***P* value**
Age ≥ 65 years	1.55 (1.18-2.05)	0.002
Female sex	0.80 (0.65-0.99)	0.040
Body mass index ≥ 25 kg/m^2	1.29 (1.07-1.57)	0.008
Hypertension	0.97 (0.78-1.21)	0.830
Diabetes mellitus	1.34 (1.10-1.62)	0.003
Previous coronary artery disease	1.23 (0.95-1.60)	0.015
Diagnosis of acute myocardial infarction	1.22 (0.97-1.52)	0.078
Number of obstructive epicardial arties	1.26 (1.11-1.42)	<0.001
Current cigarette smoking	1.23 (0.98-1.54)	0.067
Estimated GFR < 60 mL/min/1.73m^2	1.56 (1.26-1.93)	<0.001
Left ventricular ejection fraction < 55%	1.25 (1.00-1.56)	0.045
Beta-blockers	0.91 (0.78-1.15)	0.593
Renin-angiotensin system blockers	0.91 (0.75-1.11)	0.367
ePWV ≥ 11.6 m/s (cut-off value)	3.56 (2.77-4.58)	<0.001

ePWV, estimated pulse wave velocity; MACE, major adverse cardiovascular event; GFR, glomerular filtration rate; HR, hazard ratio; CI, confidence interval.

Supplementary Table S3. Multivariable analysis showing the independent association between ePWV tertile and MACE

**Independent variable**	**HR (95% CI)**	***P* value**
Age ≥ 65 years	2.04 (1.53-2.72)	<0.001
Female sex	0.78 (0.63-0.97)	0.025
Body mass index ≥ 25 kg/m^2	1.27 (1.05-1.54)	0.012
Hypertension	0.96 (0.77-1.19)	0.735
Diabetes mellitus	1.28 (1.05-1.55)	0.011
Previous coronary artery disease	1.27 (0.98-1.65)	0.065
Diagnosis of acute myocardial infarction	1.22 (0.97-1.52)	0.078
Number of obstructive epicardial arties	1.26 (1.11-1.43)	<0.001
Current cigarette smoking	1.20 (0.95-1.50)	0.111
Estimated GFR < 60 mL/min/1.73m^2	1.58 (1.28-1.95)	<0.001
Left ventricular ejection fraction < 55%	1.26 (1.01-1.58)	0.036
Beta-blockers	0.94 (0.77-1.42)	0.545
Renin-angiotensin system blockers	0.92 (0.75-1.12)	0.416
ePWV tertile		
The lowest tertile (5.50 ~ 9.89 m/s)	1	-
Middle tertile (9.91 ~ 11.98 m/s)	2.49 (1.81-3.42)	<0.001
The highest tertile (11.99 ~ 17.52 m/s)	61.8 (4.33-8.80)	<0.001

ePWV, estimated pulse wave velocity; MACE, major adverse cardiovascular event; GFR, glomerular filtration rate; HR, hazard ratio; CI, confidence interval.
